# Current climate for digital game-based learning of science in further and higher education

**DOI:** 10.1093/femsle/fny237

**Published:** 2018-09-26

**Authors:** Carla L Brown, Mary Ann Comunale, Brian Wigdahl, Sandra Urdaneta-Hartmann

**Affiliations:** 1 Department of Microbiology and Immunology, Disease, Drexel University College of Medicine, Philadelphia, PA 19102, USA; 2 Institute for Molecular Medicine and Infectious Disease, Drexel University College of Medicine, Philadelphia, PA 19102, USA

**Keywords:** science education, serious games, digital games, digital game-based learning (DGBL), learning technologies, further and higher education

## Abstract

Digital game-based learning (DGBL) is being used increasingly as an alternative learning tool to teach science in further and higher education. A variety of digital game formats currently exist for science learning, alongside diverse methods for their implementation and evaluation. This paper aims to provide a broad summary of the field by discussing the current platforms for DGBL and examples of games played on them. These include gamified simulations and traditional digital games delivered through personal computer and online software; mobile games delivered through downloaded applications for devices such as tablets and mobile phones; and educational modifications of commercial games, known amongst gamers as ‘mods’. To conclude the summary, the paper discusses the current challenges and barriers associated with DGBL in further and higher science education, and potential strategies researchers may consider to overcome them.

## Teaser

Digital game-based learning (DGBL) is an emerging trend in further and higher education. This minireview showcases current trends in DGBL and games for learning microbiology and other sciences.

## INTRODUCTION

Video games or digital games became embedded in human culture in the early seventies, establishing a digital ‘gamer’ community with the release of arcade games. Digital games like *Pizza Tycoon* and *Start Up* were recognized for their educational content; however, as these games were designed primarily for fun and entertainment, there were inherent limitations on their use as educational tools. In response to this, the educational gaming market began to grow (Prensky 2001a). Although many of the early digital games for learning were of poor quality, significant advancements made in response to scholarly inquiry and research have helped improve, redefine and reassess this novel pedagogy. Digital game technologies and platforms have also evolved over the past few decades. However, the digital game classification retains the basic requirements of game play: a rule-based competitive play (for fun) with a clear goal, resulting in variable or quantifiable outcomes that are ranked (Jaipal-Jamani and Figg 2018).

Games designed entirely for educational purposes, also known as ‘serious games’, were first acknowledged as a growing trend in higher education by the *NMC Horizon Report: Higher Education Edition* in 2005 (Johnson *et al.*^2015^). Key influencers at that time were the MIT ‘Games to Teach’ project and the research group at University of Wisconsin led by Constance Steinkuehler, PhD and Kurt Squire, PhD. They became recognized as serious game developers and game-based learning (GBL) research powerhouses. Today, interest in serious games is significant, as evidenced by the projected market growth from $3.2 billion in 2017 to $9.2 billion by 2023 (Sonawane 2017). This interest is also demonstrated in science education by an increase in scholarly research, game development and game use in many science disciplines.

This manuscript presents the current climate of digital game-based learning (DGBL) application in further (i.e. high school) in and higher (F&H) science education. A selection of games available for microbiology and other scientific fields are organized by the platform in which the game is played. To ensure a robust account is given, this paper only includes games that have been discussed in the primary literature or at scholarly events. In addition, barriers and strategies associated with DGBL in F&H education are discussed.

## HIGHER EDUCATION AS A TECHNOLOGY-DRIVEN ENVIRONMENT

The current generation of students can be considered fundamentally different from former generations due to significant changes in their media consumption behaviors. Studies have found that today's higher education students find digital technologies easy to use (Prensky 2001b; Oblinger, Oblinger and Lippincott 2005), but some counter the portrayal of today's youth as having a biologically ingrained preference or remarkable aptitude for digital technology (Selwyn 2009; Rooney 2014). On average, U.S. college students (aged 18–34) own seven technology devices, mostly laptops and smartphones, followed by video game consoles owned by two-third of the surveyed population (re:fuel 2013). Also, college students spend approximately 141.2 h/week on personal technology devices, 48.5 h on mobile phones and 40.1 hours on laptops and computers (re:fuel 2013). Furthermore, publishing giant McGraw Hill found that >80% of college students used a smartphone or a tablet to study (re:fuel 2013), making DGBL for mobile devices an attractive target.

Some higher education institutions have begun implementing DGBL as a means to increase student engagement, motivation and help achieve learning outcomes (Jonathan *et al.*^2006^; Ebner and Holzinger 2007; Warburton 2009; Bulander 2010; Bellotti *et al.*^2012^; Longstreet and Cooper 2012; Ibrahim *et al.*^2017^). Marc Prensky defined DGBL as ‘any marriage of educational content and computer games’ (p. 145) (Prensky 2001a). Adults with college experience prefer strategy games, which require higher order thinking skills indicating that college students like games that challenge them intellectually (Brown 2017). There has been a growing trend in online learning and blended learning platforms in higher education, demonstrated by their precedence in the NMC Horizon Report since 2012. Due to the ease of use of digital technology by most college students, and the overall uptake of digital games among student populations, DGBL represents a novel and relevant method to supplement and revolutionize learning for the current and future generations of F&H education students, in both online and traditional face-to-face classrooms.

## DIGITAL GAMES FOR FURTHER AND HIGHER SCIENCE EDUCATION

Science is a complex discipline and is thought to involve specific processes and components that many individuals may find difficult to remember (Driver *et al.*^1994^; Aikenhead 2006). Students may feel detached from the topics and theories that are difficult to relate to other life experiences. Lectures as a standalone pedagogical approach have been shown to be rather ineffective in the current outcomes-based environment in higher education (Aikenhead 2006). On the other hand, games are also insufficient as a standalone teaching method. Thus, it is unlikely that GBL (including digital) will replace traditional forms of instruction (lectures, textbooks, reading), especially in F&H education, but rather supplement and complement it. DGBL can provide an interactive learning experience controlled by the user that includes personalized and individual discovery, and allows learning and revision of complex content with instant feedback (Biggs 2011). Digital games aimed at F&H science education should align with higher order learning objectives (e.g. application of knowledge, critical thinking) to be as effective or better than traditional methods of teaching. There is a growing body of evidence supporting DGBL as an effective pedagogical approach in post-secondary science programs (Hainey *et al.*^2011^), yet serious games comprise a small fraction of all games produced. Not surprisingly, scholarly research on DGBL for science education is also limited and a specific strategy for its implementation or evaluation has yet to be established (Table 1).

**Table 1.** Examples of serious games for DGBL of science in further and higher education.

Game	Type	Field of science	Developer	Topic	Implementation strategy	Ref.
CD4 Hunter	Mobile Game	Virology, Infectious Diseases	Drexel University College of Medicine	HIV-1 binding and entry to CD4+ T cells	As a supplemental learning tool in undergraduate and graduate courses. Transfer of knowledge, perceived knowledge gain and enjoyment of playing the game are currently being researched. Research of faculty perceptions of the game and DGBL is also planned.	iTunes and Google Play Stores (Brown *et al.*^2016^)
Chairs!	Mobile Game	Organic Chemistry	University of Akron	Isomer conformation	Students watched a video on isomers at home and then in class received a lecture and also played the game. DGBL was assessed in the unit exam.	Winter *et al.* (2016)
Foldit	Online PC Game	Molecular biology, Biochemistry	University of Washington	Protein structure	A supplementary learning resource on protein structure with college students. DGBL was evaluated by students’ self-assessment of perceived improvement understanding of the content, and game enjoyment.	Cooper *et al.* (2010); Farley (2013)
Immune-Quest	PC Game	Immunology	Syndaus Inc.	Human immune system	Biology majors received lecture and played the game to supplement learning; pre- and post-tests covering topics in the lecture and the game were utilized to measure DGBL.	Raimondi (2016)
Kahoot!	Online Quiz Modification	Chemistry	Autonomous University of Barcelona	Adaptable, real-time quizzes and surveys	Quizzes were utilized after each lecture during college semester. DGBL was evaluated based on assessment of students mean grades	Ares *et al.* (2018)
Labster	Gamified Simulation	Biomedical Sciences (various areas)	Labster	Virtual laboratories	An educational activity in university classrooms in combination with lectures. Knowledge gain was evaluated using pre- and post-tests, and scores compared (traditional lecture only vs. combination of game and lecture).	Bonde *et al.* (2014)
Malaria Invasion	Mobile Game	Parasitology, Infectious Diseases	Drexel University College of Medicine	Red blood cell invasion by *Plasmodium sp.*	As a supplemental learning tool in undergraduate and graduate courses. Planned DGBL research: transfer of knowledge, perceived knowledge gain, and enjoyment of playing the game, and faculty perceptions of the game and DGBL is also planned.	iTunes and Google Play App in Sept. 2018. Comunale *et al.* (2018)
Meta!Blast	Gamified Simulation	Cell Biology	University of Iowa	3D structure of the cell	Installed within a science museum exhibit as an educational activity aimed at high school students. DGBL was evaluated using pre-and post-tests.	Wurtele *et al.* (2010)
Polycraft World	Vanilla Minecraft Modification	Organic Chemistry	University of Texas, Dallas	Materials science	Supplementary tool in a chemistry course for 11 weeks. DGBL was measured by students’ abilities in class and their performance in assessments.	Smaldone *et al.* (2016)
Solve the Outbreak	PC and Mobile Game	Epidemiology	CDC	Disease outbreaks	Lesson plans developed for implementation of game into high school and college classrooms. DGBL has not been evaluated.	iTunes and Google Play Stores

### Gamified simulations

**Figure 1.**
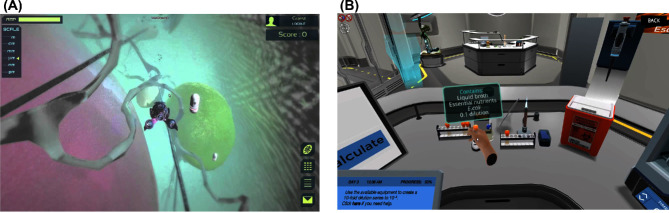
Gamified simulations for teaching cell biology and laboratory techniques. (**A**) Screenshot of Meta!Blast, a gamified simulation software that was designed to teach high school and college students about cellular biology. (**B**) Screenshot of Labster, a gamified simulation that allows students to complete a wide range of virtual labs online including medical genetics, microbiology, forensics and other laboratory techniques such as high-performance liquid chromatography (HPLC).

In F&H science education, DGBL has primarily been through PC-based gamified simulations. Simulations are ‘computational models of real and/or hypothesized situations of natural phenomena that allow users to explore the implications of manipulating or modifying parameters within the models’ (Wiggins 2016; Proulx, Romero and Arnab 2017). Virtual simulations differ from animations or other forms of 2D/3D digital media, as users are able to interact directly with the processes and components involved (Clark *et al.*^2009^). Gamification is the application of game mechanics or game elements into a non-game setting to improve player engagement or motivation (Deterding *et al.*^2011^). Simulations can be gamified by adding rewards and feedback systems (e.g. points and badges). The PC-based gamified simulation *Meta!Blast*, designed for college and high school students, is an interactive module for investigating cellular structure and function (Fig. 1). By incorporating missions that must be completed within cells, *Meta!Blast* has been shown to enable students to learn by carefully guided discovery as they navigate a cell in 3D within a dynamic metabolic environment (Plass, Homer and Hayward 2009). *Meta!Blast* provides an interactive experience and aesthetic on par with those of professionally developed commercial computer games by including immersive and high-quality artwork, an engaging storyline and unique exploratory experiences (Wurtele *et al.*^2010^). Although learning effectiveness in the higher education environment has yet to be investigated, *Meta!Blast* was evaluated using pre- and post-tests about basic cell biology concepts with ∼50 high school students while installed in a science museum exhibit. Results showed that student scores increased by 32% on average (*P *< 0.05) (Schneller *et al.*^2012^).

Another example of a gamified simulation is Labster, which has developed a series of immersive virtual laboratories (vLabs). Labster provides opportunity for open-ended investigation enhanced through gamified elements, creative storytelling and 3D visualization in >15 subjects including virology, medicine, bio-engineering, chemistry and microbiology (Fig. 1). The learning effectiveness of vLabs, which are currently used in numerous institutions including University of Glasgow, Harvard Medical School and University of Copenhagen, has been investigated in college classrooms. Two vigorous research investigations using pre- and post-test evaluation strategies concluded that the best learning outcomes are reached when vLabs are used in conjunction with traditional learning methods (*n* = 91) (Bonde *et al.*^2014^), and that a significant increase in learning is attained by students regardless of their baseline knowledge, low pretest score (*n* = 86), moderate pretest score (*n* = 101) and high pretest score (*n* = 113) (Makransky *et al.*^2016^). Interestingly, researchers also found that intrinsic motivation was significantly increased in the student population with moderate or high pre-test scores, but was not significant in those with low baseline knowledge (Makransky *et al.*^2016^). Yet, those with low pre-test scores showed the greatest increase in learning. These studies provide a strong argument for using gamified vLab simulations to supplement learning.

### Traditional digital computer games

**Figure 2.**
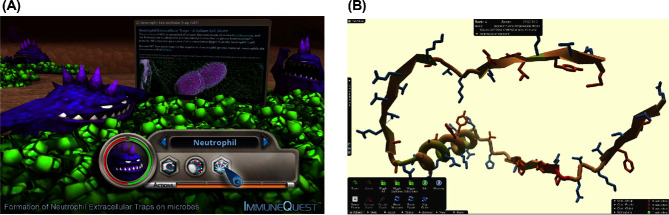
Digital science games developed to teach immunology and protein chemistry. (**A**) Screenshot of ImmuneQuest, a 3D PC game designed to teach students about immunology in the human body. (**B**) Screenshot of Foldit, an online puzzle game that allows player to input to research by solving protein structures.

Many educators are investigating their own skills in game design and development. *Foldit* is an online multiplayer PC game that allows players to collaborate and compete, individually or in teams, to solve protein structure puzzles (Cooper *et al.*^2010^; Khatib *et al.*^2011^) (Fig. 2). *Foldit* was designed as a ‘citizen science game’ that made use of crowdsourcing strategies to gain public and gamer input into scientific research. One of its greatest success stories is the solving of a retroviral protease crystal structure known as M-PMV (Khatib *et al.*^2011^). *Foldit* was investigated as a supplementary learning resource for college students studying biochemistry in an agro-ecotechnology program (Farley 2013). All students reported perceived improvement in understanding of this topic, albeit it was a small sample (*n* = 25). Additionally, 94% reported that the game was ‘fun to play’ and 47% stated that they had played the game outside of class, suggesting intrinsic motivational properties of DGBL. Further work that includes measurement of baseline knowledge is required to determine the effectiveness of *Foldit* as an educational tool in this setting.

*ImmuneQuest* is a digital game that supplements immunology instruction by allowing students to build and control a virtual immune system. In *ImmuneQuest*, correct answering of immunology questions allows players to ‘level up’ their immune cells. Its learning effectiveness was assessed in a small cohort of biology majors (*n* = 20) enrolled in an immunology course using pre- and post-learning questionnaires completed at the start and end of the module (Raimondi 2016). Although learning questionnaires reported that topics covered specifically by *ImmuneQuest* improved from 32% to 98% (*P* < 0.05), further work is required with a larger cohort to determine its efficacy as an educational tool. Some study participants found the game frustrating or that their focus was simply on beating the game rather than learning. However, others found the game provided useful visuals and reinforced course material.

Digital games designed for the purpose of public engagement and education of a broad audience are also being utilized for F&H science education. *Solve the Outbreak*, a problem-solving game, was developed by the Center for Disease Control and Prevention (CDC) to communicate key epidemiology concepts. In addition, the CDC developed formal lesson plans to aid implementation in classrooms. This game has been utilized by lecturers at Drexel University College of Medicine as a way to introduce DGBL and spark discussion in infectious diseases courses. Briefly, students received a lecture on DGBL, played Solve the Outbreak, and then critiqued the game based on scientific content, game design and relevance for higher science education (Fred Krebs, PhD, pers. comm., January 15, 2018). Dr Krebs reported that most students suggested continued use of Solve the Outbreak in the course. Originally developed as a web PC game, *Solve the Outbreak* is also available on iOS and Android mobile devices to align with modern technologies but there are no published studies assessing the effectiveness of this game.

### Mobile games

Despite the ubiquitous use of mobile and touchscreen devices, there is a dearth of science-based mobile games developed specifically for F&H education. Mobile games represent an ideal platform for science learning due to the high uptake of ‘apps’ in the daily lives of current F&H education students. Several iTunes and Android mobile game apps developed by non-educational groups have integrated scientific concepts within core game mechanics. For example, *Plague Inc.* (Ndemic Creations) is a highly successful strategy game in which players take the role of a pathogenic microorganism and infect the global population (Vaughan 2017). *Pandemic* (Asmodee Digital), a digital board game that focuses on epidemiology and infection control through player team work (Wallace *et al.*^2012^), and *Superbugs* (Nesta), which communicates the problem of antimicrobial resistance to the general public. Robinson, Turner and Sweet (2018) discuss strategies for using entertainment games such as *Plague Inc.* to teach the concepts of disease epidemics and pandemics, to facilitate class discussion, or as a means for students to develop and test a scientific approach in real time. Although commercial games of this format are useful for introducing scientific concepts to students, the primary purpose of these games is entertainment with educational benefit being secondary. To date, there are no formal studies published that show learning was increased by incorporating *Plague Inc.* into the learning module.

**Figure 3.**
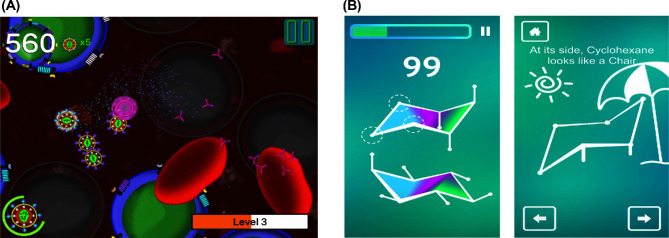
Mobile games for teaching microbiology and organic chemistry in higher education. (**A**) Screenshot of CD4 Hunter, an educational mobile game about how HIV-1 infects CD4+ T cells in the human body. (**B**) Screenshot of Chairs!, a mobile game to teach about isomer conformation and alkanes.

A recent example of a mobile game designed specifically for use in microbiology, virology and infectious disease undergraduate and graduate courses is *CD4 Hunter^TM^*, developed by the Department of Microbiology and Immunology and Institute for Molecular Medicine and Infectious Disease at Drexel University College of Medicine (Brown *et al.*^2016^). *CD4 Hunter* was designed to educate players about the human immunodeficiency virus (HIV-1) replication cycle and focuses on the binding and entry of the virus into CD4^+^ T cells (Fig. 3). In the game, the player navigates the bloodstream as HIV-1 virions and searches for CD4^+^ T cells that possess a CD4 receptor and the specific co-receptor. CD4 Hunter is available on iTunes and Google Play App stores and is currently being implemented to supplement courses taught at Drexel University College of Medicine. Research is ongoing to evaluate the effectiveness of CD4 Hunter as a learning tool for undergraduate and graduate students (Comunale and Urdaneta-Hartmann 2018). This same research group also developed *Malaria Invasion™*, an iOS and Android mobile game about the molecular mechanisms by with *Plasmodium* invades red blood cells. It is targeted for implementation in MS and PhD programs in microbiology and infectious disease (Comunale *et al.*^2018^).

*Chairs!* (Fig. 3) is a problem-solving based mobile game that teaches about conformational analysis of alkanes in organic chemistry (i.e. the flip ring of cyclohexene). It requires players to correctly draw bonds at the right angles and axis on different conformational isomers of cyclohexane (Winter, Wentzel and Ahluwalia 2016). Red colored bonds have been used to denote incorrect angles and axis, and green bonds represent correct conformations. *Chairs!* has been made available on iTunes and Google Play app stores and has been formally assessed in both high school and college classrooms (Winter, Wentzel and Ahluwalia 2016). Second-year organic chemistry college students (*n* = 50) learning about conformational analysis watched a video on isomers at home and then in class received a lecture and also played *Chairs!* for 30 min in groups, and then competed with peers to achieve the highest score. The potential learning efficacy was indicated in the unit exam where the average grade was 71% and students earned an average grade of 82.5% on questions associated with cyclohexane, an alkane featured in *Chairs!*. In addition, 90% of students reported that playing *Chairs!* helped them better understand the concepts (Winter, Wentzel and Ahluwalia 2016).

### Modifications or ‘mods’ of commercial games

**Figure 4.**
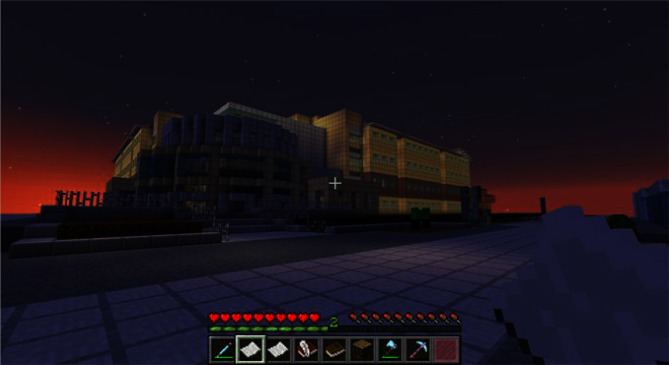
‘Modding’ existing game software for teaching of chemistry in higher education. (**A**) Screenshot of Polycraft World, a ‘mod’ of Minecraft, designed to teach college students about organic chemistry and distillation.

The current era of gaming has been marked by complex, multiplayer and multiworld games that have provided researchers with excellent platforms to build upon to develop educational software. Indeed, rather than construct games ‘from scratch’, researchers may prefer to modify or adapt an existing game or software and incorporate educational content to achieve science learning. The adaptation of an existing commercial game software is known as ‘modding’ (Kow and Nardi 2010). A recent example of this for F&H science education is *Polycraft World*, a modification or ‘mod’ of *Minecraft* developed by researchers at the University of Texas, Dallas (Smaldone *et al.*^2016^). *Polycraft World* is played via an online PC server. This server is often termed ‘*Vanilla Minecraft’* because it allows modifications while retaining original *Minecraft* game mechanics (Fig. 4). In accordance with the original *Minecraft* game mechanics, *Polycraft World* provides a platform to develop and fabricate materials in their virtual world that are related to organic chemistry. The synthesis of new materials and items in *Polycraft World* requires application of chemistry knowledge (e.g. materials texture, understanding the effects on aromatic ring substitution reactions, etc.). Preliminary research showed that this game may be an effective educational tool for teaching college-level organic chemistry. After playing the game for 11 weeks, 8/13 chemistry students were able to correctly draw two or three levels of a crude oil distillation tree, a key component of the core game mechanics; 17/26 students were able to correctly identify LDPE, a common polymer used in the game, as low-density polyethylene (Smaldone *et al.*^2016^). However, a limitation of the study is the lack of a control group.

*Kahoot!* is a simple-to-use online tool that allows educators to create and deploy gamified customized, interactive quizzes and surveys, accessible by students on their own mobile devices or computers. Players compete against each other in real time for high scores, before the timer built into the game runs out. *Kahoot!* is mostly used in K-12 education but some have modified it for F&H education. Its effectiveness was investigated in a college chemistry course in which students completed a *Kahoot!* quiz on their smartphones either after their lectures (Group 1; *n* = 42) or after every two lectures (Group 2; *n* = 47) (Ares *et al.*^2018^). Their academic performance was compared with that of students who completed the course in the previous year in which *Kahoot!* was not used. This pilot study showed that students in both *Kahoot!* groups had better grades and a higher pass rate than students that completed the course in the previous year. Furthermore, the improvement was more prevalent among students who performed the best in the *Kahoot!* quizzes. Also grades were better when *Kahoot!* was played more frequently (Group 1). The researchers concluded that *Kahoot!* benefited the students and enhanced the course, but acknowledged that more research is needed to confirm the results.

## CHALLENGES AND STRATEGIES ASSOCIATED WITH DGBL IMPLEMENTATION

It is widely accepted in the literature that there are many barriers to implementing DGBL in F&H education, including complex technology and skills needed to develop digital games; the small number of peer-reviewed publications; no standardized framework or policy for use or assessment of games; limited amount of time, pedagogical training, and digital literacy of faculty; and limited resources of departments and institutions (de Freitas *et al.*^2013^; Comunale 2017). Yet, early adopters of DGBL in F&H education have found ways to circumvent the many conflicts by developing or adopting existing digital games, conducting DGBL studies, publishing and organizing conferences in the hopes of sharing knowledge and providing opportunities to network and grow the field. Below are suggested resources faculty and institutions can research to learn more about DGBL and help determine if it is suitable for them and their students.

### Peer-reviewed literature for DGBL

Research of DGBL in higher science education has increased, as evidenced by a systematic search for publications in Google Scholar for ‘digital games, higher science education’ results per year (2010: 23 100 results; 2015: 34 000 results; 2017: 35 100 results). In addition, indexed, peer-reviewed journals within the field have emerged within the last decade or so, and in particular within the last 5 years, such as *International Journal of Computer Games Technology*, established in 2007; *International Journal of Game-Based Learning*, established in 2011; *International Journal of Mobile Learning and Organization*, established in 2013.

Increasing scholarly work is an effective approach to establish and gain acceptance of best practices for serious game development and assessment. A wide range of grassroots evaluation methods to assess the learning effectiveness of complex science using DGBL can be used in combination or individually. They include the use of pre- and post-test knowledge assessments; post-test only used with an additional control variable (i.e. students play an unrelated game); measurement of skillsets gained after implementation of DGBL and comparison of student mean grades with control populations, and qualitative interview or focus group research designs. Although grassroots methodologies may suffice for internal funding opportunities and to determine implementation strategies in academic programs, they are not enough to demonstrate the full potential of DGBL for science learning in F&H education. Admittedly, the scarcity of digital games for higher or further science education impedes the development of a best practice model for assessment.

### Conferences and serious games competitions

To complement the increase in scholarly journals and peer-reviewed literature, there has been an increase in regional, national and international conferences and events for GBL that provide valuable professional development opportunities in all areas, including DGBL. The conferences include specialized tracks and workshops, including game design and development, implementation and assessment strategies, creativity, motivation, tabletop and digital games, and tracks that are specific to academic levels and disciplines and some host concurrently game competitions. Some conferences have specific science/healthcare/medical tracks (Table 2). Thus, conferences provide an unparalleled learning experience for educational game designers, developers and implementers. Participants have an opportunity to network and showcase their games, giving attendees a chance to learn through playing games, while also providing developers with valuable and immediate feedback. In addition, many scientific conferences, such as the American Society for Microbiology (ASM) Conference for Undergraduate Education (ASMCUE) and ASM’s Microbe, include sessions that focus on teaching and learning technologies, including digital games and applications.

**Table 2.** Select serious games conferences with relevance to further and higher education and science.

Conference	Established	Webpage URL
North American Gaming and Simulation Association Conference	1962	http://www.nasaga.org/
International Society for Technology in Education (ISTE)*	1979	https://www.iste.org
International Conference on Game-Based Learning and Serious Games	1998	https://waset.org/conference/2018/04/boston/ICGBLSG
Digital Games Research Association (DiGRA) Conference	2003	http://www.digra.org/conference
Games for Change/VR for Change*	2004	http://www.gamesforchange.org
European Conference on Games Based Learning (ECGBL)*	2006	http://academic-conferences.org/ecgbl/ecgbl-home.htm
Meaningful Play Conference	2008	http://meaningfulplay.msu.edu
Serious Play Conference*	2010	http://seriousplayconf.com/
CUNY Games Conference	2014	https://games.commons.gc.cuny.edu/
Games and Learning Association	2012	https://conf.seriousgamessociety.org
International Conference on Serious Games and Applications for Health	2012	http://www.segah.org
Games and Learning Alliance Conference (GALA)	2012	https://conf.seriousgamessociety.org/
Gotland Game Conference	2018	http://gotlandgameconference.com

An asterisk designates that it hosts a concurrent game competition.

### Professional and career development opportunities

In the university environment, new interdisciplinary programs are also being offered that train scientists in media, game design, journalism and communication. Since 2016, Drexel University College of Medicine offers an advanced degree in Biomedicine and Digital Media, which intersects education and training in infectious disease and biomedical technology with game design and interactivity. Similarly, the department has designed fellowships and internship opportunities for postdoctoral scientists, graduate and undergraduate students interested in this emerging field. In addition to providing career training for students, professional development for faculty has also been under investigation. Game Changers Open Course at the Disruptive Media Lab, University of Coventry (funded by Higher Education Funding Council; Catalyst Call for Innovative Learning), is an online course based on the principle of educational game design for higher education. The key aim of the course is cited on the university website, as ‘facilitating new models of teaching and learning, new methodologies in interdisciplinary and cross faculty collaboration to make game design and development more culturally open and accessible to staff and students.’ Indeed, based on the increased training and educational diversification that has taken place in the DGBL arena, the number of alternative careers has notably expanded in the science and academic tracks. This was showcased in a recent Nature Careers article that focused on the prevalence of scientific researchers exploring roles and projects in game design and development (Kwok 2017). To this end, further progression in this field will also require increased funding opportunities to support development of new games and innovative projects for distinct science subjects.

### Funding for DGBL research and development

Funding specific for DGBL research and development in F&H education is not as abundant. However, several opportunities exist to encourage innovative education strategies of science in F&E education. In the United States, The National Science Foundation has various funding mechanism to innovate undergraduate and graduate education (National Science Foundation (NSF) 2018a,b). Other funding opportunities include the UK Games Fund that awards seed funds for game projects with social or educational benefit (UK Games Fund 2018); the National Institute for Health Research has offered several funding awards for STEM-related software or games as part of their 5-year Federal CoSTEM Strategic Plan in the priority areas of ‘games for learning’. In addition, The Bill Gates Foundation recently invested $2.6 million to Quest Atlantis for developing digital games that educate in math, literacy and science (Bill and Melinda Gates Foundation 2011).

## CONCLUSION

Implementation of digital games for science learning is no simple task. At minimum, it requires access to relevant software, knowledge on digital technologies and pedagogical expertise. However, as shown by this manuscript, educators are creatively overcoming these challenges to drive the implementation of DGBL in F&H science education through scholarly research and creation of networking opportunities. Overall, this manuscript reported use of various game formats and platforms, indicating that educators are striving to innovate the field rather than use a single method of implementation. This manuscript reported DGBL implementation in the form of engaging mobile games (often developed in-house), modifications of commercially relevant software (e.g. Minecraft) and use of immersive simulations that allow students to experience complex science on their own devices. Indeed, the increasing use of commercially relevant games and software for DGBL indicates a significant role for interactive and engaging learning technologies in future science learning.

However, although innovation was reported extensively for digital game resources, this trend was not as widely reported for assessment and evaluation methods. In the current climate, a wide variety of grassroots evaluation strategies are being adopted that may be hindering researchers determining the true benefit and impact of DGBL for science education. Most of the research has also been conducted in small cohorts. Robust evaluation of DGBL may require the development of a consensus methodology adopted by all researchers. Knowledge gained through research should be disseminated through peer-reviewed publications, conferences and networks reported in this manuscript. Ultimately, there is great opportunity for researchers to further develop the field of DGBL of science through professional development, collaboration, knowledge exchange, and use of available resources and games.

Due to inherent limitations of DGBL (e.g. it may be hard to develop games that can teach all learning objectives for a specific topic only through gameplay), it is unlikely to replace traditional forms of instruction such as lectures and reading textbooks and primary literature. Thus, games may be insufficient as standalone teaching methods. However, games can supplement and complement traditional forms of teaching and learning.

## DISCLOSURE

All images are used with permission. CLB is Founder of Game Dr. Limited (Edinburgh, UK) a private limited company that develops science mobile games for universities and academic research groups in the United Kingdom.

## FUNDING

CLB, MAC and SUH were supported by professional development funds provided by Drexel University College of Medicine's Department of Microbiology and Immunology and the Institute for Molecular Medicine and Infectious Disease. Also, SUH was supported by a Drexel University College of Medicine Professional Enrichment and Growth (PEG) Grant, and by Research Co-Op Funding awarded by Drexel University's Office of the Provost and the Steinbright Career Development Center.

***Conflict of interest.*** None declared.
